# Structured reporting of head and neck ultrasound examinations

**DOI:** 10.1186/s12880-019-0325-5

**Published:** 2019-03-27

**Authors:** Benjamin P. Ernst, Mohamed Hodeib, Sebastian Strieth, Julian Künzel, Fabian Bischof, Berit Hackenberg, Tilmann Huppertz, Veronika Weber, Katharina Bahr, Jonas Eckrich, Jan Hagemann, Matthias Engelbarts, Matthias F. Froelich, Philipp Solbach, Richard Linke, Christoph Matthias, Wieland H. Sommer, Sven Becker

**Affiliations:** 1 Department of Otorhinolaryngology, University Medical Center Mainz, Langenbeckstraße 1, 55131 Mainz, Germany; 2 Department of Radiology, LMU University Hospital, Marchioninistraße 15, 81377 Munich, Germany; 3 Department of Gastroenterology, Hepatology and Endocrinology, Hannover Medical School, Carl-Neuberg-Straße 1, 30625 Hannover, Germany; 4 Department of General and Visceral Surgery, University Hospital Frankfurt, Theodor-Stern-Kai 7, 60590 Frankfurt, Germany

**Keywords:** Structured reporting, Ultrasonography, Head and neck Cancer, Salivary gland diseases, Lymphadenopathy

## Abstract

**Background:**

Reports of head and neck ultrasound examinations are frequently written by hand as free texts. Naturally, quality and structure of free text reports is variable, depending on the examiner’s individual level of experience. Aim of the present study was to compare the quality of free text reports (FTR) and structured reports (SR) of head and neck ultrasound examinations.

**Methods:**

Both standard FTRs and SRs of head and neck ultrasound examinations of 43 patients were acquired by nine independent examiners with comparable levels of experience. A template for structured reporting of head and neck ultrasound examinations was created using a web-based approach. FTRs and SRs were evaluated with regard to overall quality, completeness, required time to completion, and readability by four independent raters with different specializations (Paired Wilcoxon test, 95% CI) and inter-rater reliability was assessed (Fleiss’ kappa). A questionnaire was used to compare FTRs vs. SRs with respect to user satisfaction (Mann-Whitney U test, 95% CI).

**Results:**

By comparison, completeness scores of SRs were significantly higher than FTRs’ completeness scores (94.4% vs. 45.6%, *p* < 0.001), and pathologies were described in more detail (91.1% vs. 54.5%, *p* < 0.001). Readability was significantly higher in all SRs when compared to FTRs (100% vs. 47.1%, *p* < 0.001). The mean time to complete a report, however, was significantly higher in SRs (176.5 vs. 107.3 s, *p* < 0.001). SRs achieved significantly higher user satisfaction ratings (VAS 8.87 vs. 1.41, *p* < 0.001) and a very high inter-rater reliability (Fleiss’ kappa 0.92).

**Conclusions:**

As compared to FTRs, SRs of head and neck ultrasound examinations are more comprehensive and easier to understand. On the balance, the additional time needed for completing a SR is negligible. Also, SRs yield high inter-rater reliability and may be used for high-quality scientific data analyses.

## Background

Over the past decades, reports of head and neck ultrasound examinations have been written as free texts. Even today, many reports are written by hand [1–3]. Within the last few years structured reports (SR) have been advocated by various medical societies because clinical studies provided evidence for the superior nature of SRs, i.e. improvement of overall report quality, accuracy and detail when compared to free text reports (FTR) [4–9]. In addition, both the examiner and the referring clinician often have a preference for SRs in these studies due to higher levels of accuracy and clarity [10–14]. This may result in a better understanding of the pathology and its therapeutic implications [15, 16]. A healthcare professional using a SR is less likely to omit important structures. As a result, SRs are more thorough, especially when written by inexperienced professionals [13, 17]. Due to their standardized structure SRs may also be used for high-quality scientific data analyses [18].

Regardless, clinicians are often concerned that structured reporting templates are inflexible and adaption to specific findings may be imprecise and time-consuming [19, 20]. However, especially clinical examinations that follow a clearly defined workflow do benefit from a more structured approach to reporting. This includes ultrasound exams of the head and neck for evaluation of cervical lymphadenopathy, salivary gland disorders and head and neck cancer [21–23]. Additionally, there is a general lack of guidance in the use of technical terms and report structure in this field, leading to great variability in report content [1, 24]. Therefore, establishing a standard for ultrasound reports using structured reporting may be greatly beneficial for physicians acquiring ultrasound skills as well as for the referring clinician [25, 26]. The aim of the current study was to evaluate overall report quality, comprehensiveness, time needed to complete, readability and especially inter-rater reliability and clarity of template-based SRs vs. FTRs.

## Methods

### Study design

The scope of this study was to compare FTRs to SRs of ultrasound examinations of the head and neck. Physicians of our department were divided into two groups with matching experience in head and neck ultrasound. The first group (*n* = 4) used FTRs, while the second group (*n* = 5) used SRs. Subsequently, 43 consecutive patients requiring an ultrasound examination were identified in our outpatient clinic. After informed consent had been obtained, every patient was examined by two independent physicians with equal experience in head and neck ultrasound. Both SRs (*n* = 43) and FTRs (*n* = 43) were created (*n* = 86 reports). To reduce inter-observer bias in report quality, residents were not supervised by the responsible senior physician while creating the reports used within the study. See Table 1 for further patient demographics and sample characteristics.

**Table 1:** Patient demographics and sample characteristics

Characteristics	Value
Number of patients	43
Age (mean ± SD)	58.6 ± 14.8 years
Age (range; years)	20–83 years
Gender	male: 55.8%, female: 44.2%
Indication for ultrasound	Tumor follow-up: *n* = 26 Cervical lymphadenopathy *n* = 10 Salivary gland disease: *n* = 7

Indications for head and neck ultrasound consisted of scheduled follow-ups for squamous cell carcinomas of the head and neck (*n* = 26), cervical lymphadenopathy (*n* = 10) as well as parotid and submandibular salivary gland diseases (*n* = 7)

### Sample size calculation

As described by others, the number of patients needed was calculated based on the anticipated effect size when comparing the percentage of FTRs with 80% completeness or higher to SRs [27]. We estimated that 55% of FTRs would have a completeness of 80% or higher, taking into account the report quality of other imaging techniques within the literature [13, 27]. In addition, we assumed that 70% of SRs would have a completeness of 80% or higher. The power was set at 80% and the significance level was set at α = 0.05. Using these parameters, the minimum number of patients was determined, resulting in *n* = 82 (41 patients in each group) [28].

### Image acquisition

Images were acquired for all patients using a LOQIQ E9 ultrasound unit (GE Healthcare, Little Chalfont, United Kingdom) with 9 to 15 MHz linear transducers, depending on the anatomy of the patient. A web-based picture archiving and communication system (PACS, Sectra AB, Linköping, Sweden) was used to store and review acquired images.

### FTR and SR

**Fig. 1:**
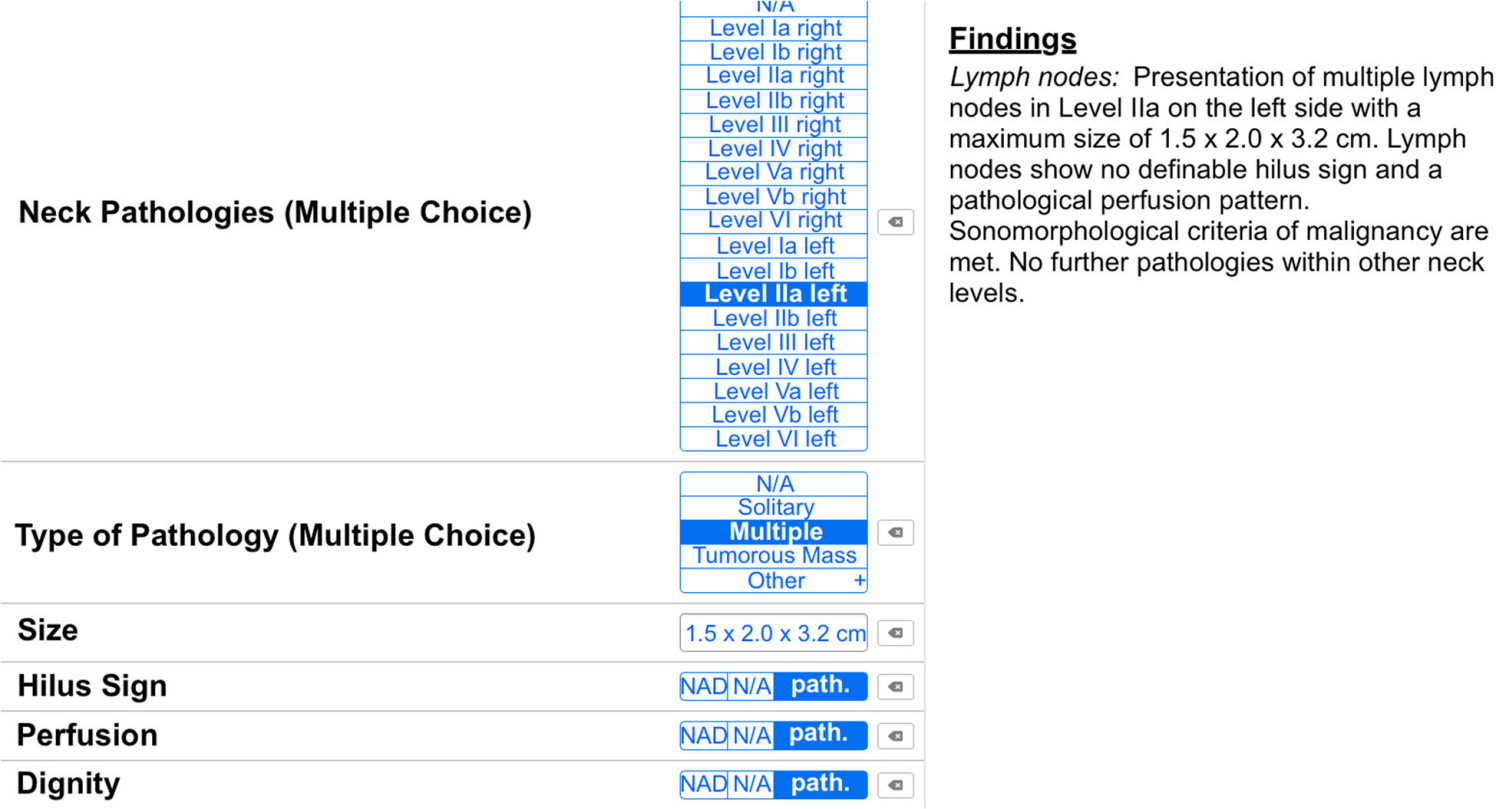
Screenshot of the decision-tree within the reporting software. Shown is an exemplary report of a lymph node pathology. On the left side, the examiner can select the corresponding neck level, number and size of affected lymph nodes as well as pathological feature such as hilus sign, perfusion pattern and assessment of dignity while the template generates full semantic sentences on the right side

The control group used the departmental standard FTR template, which is to be completed by hand. For the SR group a web-based software (Smart Reporting GmbH, Munich, Germany, https://www.smart-radiology.com/de/) was used to design a specific template for structured reporting of head and neck ultrasound examinations. The template was created in cooperation with board-certified radiologists and otorhinolaryngologists with proficiency in ultrasound examinations. The utilized medical and linguistic content is in accordance with the most recent recommendations of the German Society for Ultrasound in Medicine (DEGUM) for reported structures and terminology. The template was designed to cover all common head and neck pathologies. Examiners are guided through clickable decision-trees. Within this process, the software generates full semantic sentences from previously defined text phrases that do not require any further editing (see Fig. 1). Each and every report follows the same structure. To ensure a high degree of flexibility or to add additional comments, which are not inquired by the template, free text elements may be added at the discretion of the examiner. Furthermore, specific instruction manuals and tutorials can be integrated into the template to reduce the likelihood to consult further medical literature during reporting [29]. All reports were compiled by the examiner immediately following the examination.

### Report evaluation

Work experience and time needed to complete the report were documented during report generation. The 86 anonymized reports (43 FTRs and SRs each) were independently evaluated based on overall completeness (i.e. reporting of bilateral neck levels, salivary glands and major blood vessels), detail, readability and inter-rater reliability by one board-certified radiologist, one otorhinolaryngologist, one internist and one visceral surgeon. A specifically designed evaluation form was created by three highly experienced sonographic examiners (i.e. DEGUM Level II head and neck) for assessment. Overall report quality was defined as the combination of report completeness, detail and readability (insufficient: 0–20%, poor: 20–40%, moderate: 40–60%, high: 60–80%, very high: 80–100%). Readability was subjectively evaluated using a five-point scale (0: insufficient readability, 5: very good readability).

Additionally, we developed a questionnaire for the nine examiners. Using a ten-point visual analogue scale (10: Complete agreement, 0: Complete disagreement), participating physicians were asked about practicability (question 1), usefulness in everyday practice (question 2), improvement in report-quality (question 3), time-wise efficiency and economy (question 4), justification of additional time needed (question 5), benefits for inexperienced physicians learning ultrasound examinations (question 6) and reporting (question 7), usability by intuition (question 8) and clarity of arrangement of the template (question 9).

### Statistical analysis

Data are presented as the mean ± standard deviation. A *p*-value of less than 0.05 was considered to be statistically significant. Wilcoxon signed-rank test for paired nominal data was used to test for significance regarding completeness, detail and time required. Due to the non-parametric distribution, Wilcoxon-Mann–Whitney U test was used to compare questionnaire results. Linear regression analysis was applied to determine correlations. Fleiss’ kappa was used to evaluate inter-rater reliability [30, 31]. All statistical analyses were performed using SigmaPlot 12 (Systat Software, Inc., San Jose, CA, USA).

## Results

### Report analysis

A total of 86 reports (*n* = 43 for FTRs and SRs each) were eligible for analysis. SRs showed a significantly higher overall completeness (*p* < 0.001). Raters were able to extract information about 94.4% of previously defined structures needed within reports while FTRs yielded only 45.6%. In detail, SRs achieved higher ratings in completeness with respect to lymph nodes (96.7% vs. 46.8%, *p* < 0.001), salivary glands (95.3% vs. 88.6%, *p* = 0.002) and major blood vessels (87.5% vs. 18.2%, *p* < 0.001). Additionally, pathologies were described in significantly greater detail using the recommended terminology in SRs (91.1% vs. 54.5%, *p* < 0.001).

Mean time needed to complete the report was significantly higher using SRs (176.5 s vs. 107.3 s, *p* < 0.001).

SRs yielded significantly higher readability ratings (100% vs. 47.1%, *p* < 0.001) when compared to FTRs resulting in better information extraction and rater’s satisfaction.

**Fig. 2:**
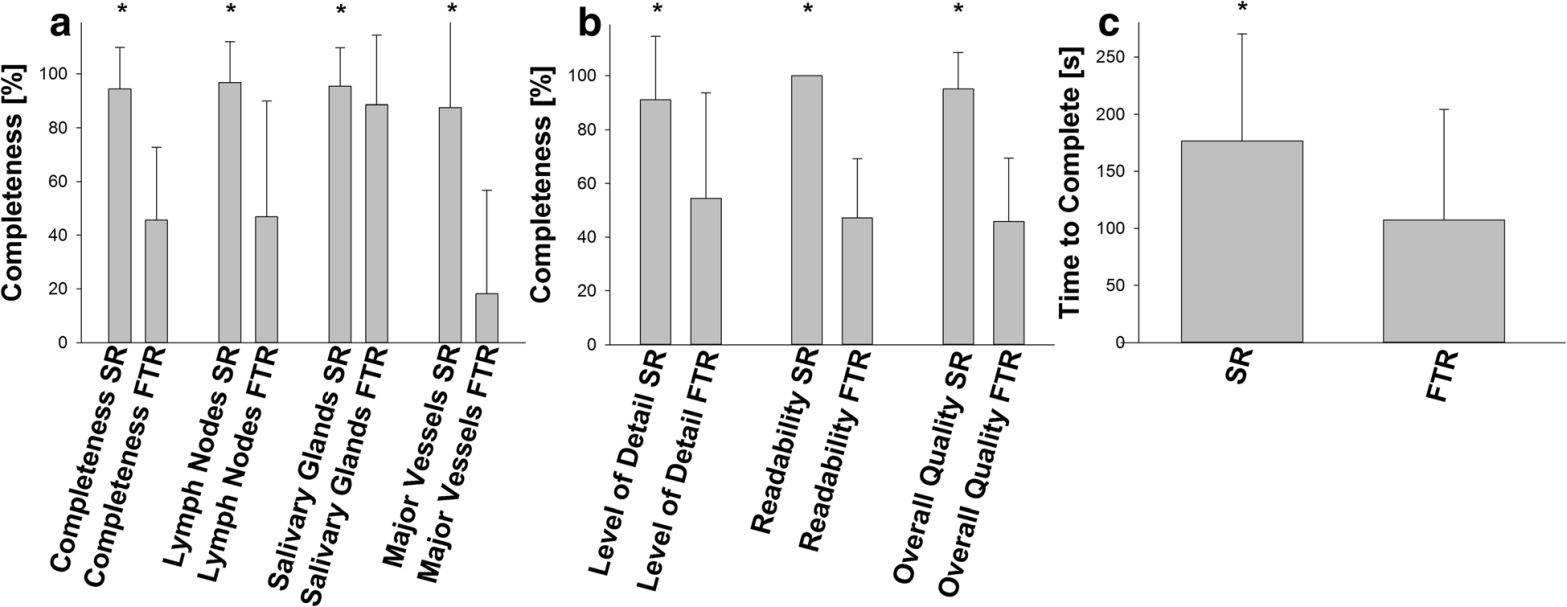
Results of report analysis. Reports were evaluated by four independent internal and external raters of different specialties. Structured reports (SR) yield significantly higher completeness rates in describing cervical lymph nodes, salivary glands and major neck vessels than free text reports (FTR) resulting in a significantly increased overall completeness (**a**). Additionally, level of detail, readability and overall report quality was significantly improved when using SRs (**b**). Time needed to complete the report was significantly shorter when using FTRs (**c**). Results are presented as mean with standard deviation. * *p* < 0.05

Consequently, overall report quality was determined and reports categorized as described above. Mean overall report quality was significantly higher in SRs when compared to FTRs (95.1% vs. 45.8%, *p* < 0.001). Insufficient to moderate report quality was significantly associated with FTRs (59.9% vs. 2.3%, *p* < 0.001) while high to very high report quality was significantly associated with SRs (97.7% vs 40.1%, *p* < 0.001). Additionally, there was no significant correlation between the time needed to complete the report and the overall report quality (R = 0.04, R^2^ = 0.038, *p* = 0.006). A detailed report analysis is shown in Fig. 2. Inter-rater reliability of SRs was very high with a Fleiss’ kappa of 0.92.

### User contentment

**Fig. 3:**
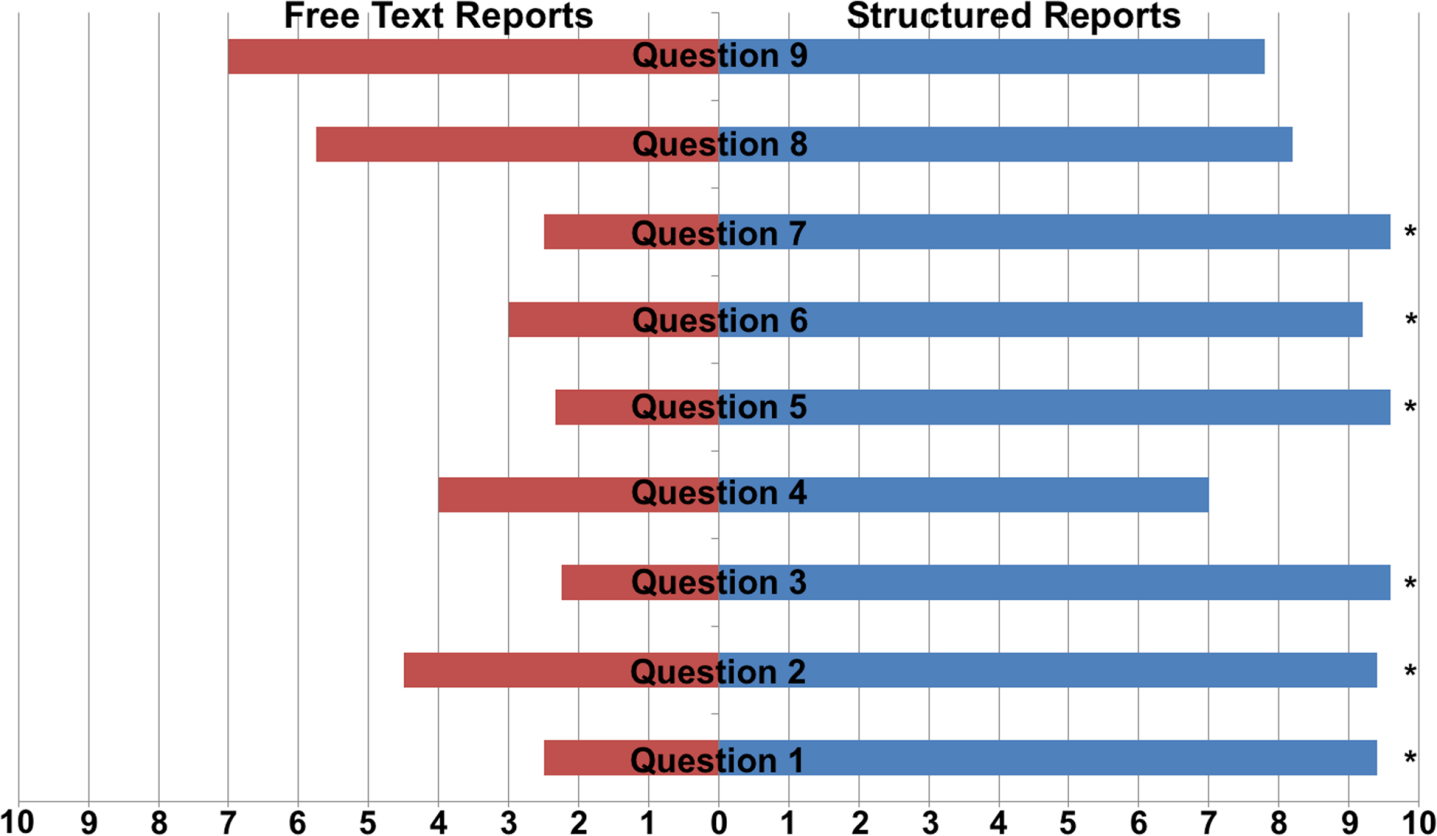
Visual analog scale (VAS) of questionnaire findings. User contentment of the nine participating examiners was evaluated using a questionnaire. VAS (10: Complete agreement, 0: Complete disagreement) shows that structured reports (SR, right side, blue bars) are regarded as practicable (question 1), useful (question 2), to improve report-quality (question 3), to be time-efficient, to have a good time-wise economy (question 4), that additional time needed may be justified (question 5), that inexperienced physicians learning ultrasound examinations (question 6) and reporting (question 7) benefit from SR, that usability by intuition (question 8) and clearness of arrangement are substantial (question 9) when compared to free text reports (FTR, left side, red bars). * *p* < 0.05

The questionnaire revealed a significant preference for SRs by all interviewed examiners (8.87 vs. 1.41, *p* < 0.001). Structured reporting was regarded as applicable for everyday use in a university medical center outpatient clinic (9.47 vs. 0.74, *p* < 0.001) and as time-efficient (8.3 vs. 3.29, *p* = 0.002). In addition, SRs were regarded as a suitable assistance for physicians unexperienced in performing head and neck ultrasound examinations in both conducting the examination (9.2 vs. 3.0, *p* = 0.016) and creating the report (9.6 vs. 2.5, *p* = 0.016). Thus, structured reporting was assumed to lead to a higher level of report quality (9.6 vs. 2.25, *p* = 0.016). A detailed analysis of questionnaires is shown in Fig. 3.

## Discussion

Head and neck ultrasound examinations are the clinical standard in routine outpatient examinations for various neck pathologies, including follow-ups for head and neck cancer patients and surgical planning [21–23, 32]. Besides a thorough examination, accurate reporting plays an important role in ensuring the highest standards in diagnostics and therapy. While conventional FTRs tend to exhibit low intra- and inter-rater reliability in terms of report quality, comparability and level of detail, structured reporting has evolved as a new promising approach in report generation [1, 11].

The aim of this preliminary, prospective single center study was to evaluate the impact of SRs of head and neck ultrasound examinations upon overall quality, completeness, detail, readability as well as time-efficiency and user satisfaction. To the best of our knowledge there have been no previous prospective studies on SRs of head and neck ultrasound examinations. Additionally, this has been one of the largest prospective studies on structured reporting in general [10–13, 33, 34]. Our data showed that the use of SRs leads to significantly improved report quality, completeness and readability. In addition, pathologies were described in significantly greater detail and users were significantly more satisfied. On the other hand, the time needed to complete SRs was significantly higher than for FTRs. These findings are consistent with those of previous studies, which have shown a superior report quality of SRs in a number of diagnostic modalities [10–13, 27]. Additionally, there is a significant preference for SRs by both the examining and referring physicians, due to its standardized approach and conformity with clinical standards and guidelines [14].

Furthermore, SRs of head and neck ultrasound examinations may also be of educational value for young residents [13]. Head and neck ultrasound represents a complex examination technique due to the structural complexity of this particular anatomic region. Besides, the use of a structured template may have an educational value by guiding the inexperienced resident through the examination and pinpointing key structures. This hypothesis is supported by various publications that were able to show a reduction of missed pathologies [8, 19, 35]. Therefore, SRs are associated with improved diagnostic accuracy and comparability.

A controversial topic in medical reporting is whether SRs provide settings that are too rigid. This is supported by various publications that were able to demonstrate non-inferior to superior report quality generated by FTRs [2, 19, 20]. Furthermore, SRs have been associated with a lack of linguistic quality, phrasing and terminology. These problems may be addressed through careful planning. It appears essential to use standardized and recommended language, which should be discussed in advance by examining and referring physicians to ensure a high level of consensus and consequently report quality [36]. Advanced computer technologies may be a key to overcoming problems with inflexibility and inferior linguistic quality by facilitating intelligent decision trees. Furthermore, crosslinking possibilities within the template and the possibility to add free text elements ensure a high degree of completeness. In accordance with the literature, there were no problems associated with the use of free text elements in order to add details to the report [10, 37]. Once a template with no grammatical or orthographical mistakes is implemented, especially SRs generated by non-native speakers might yield a higher report quality than FTRs. While other studies were able to show that structured reporting tends to be time-saving, our data demonstrate a significantly longer time to complete the report when compared to FTRs [19, 20, 37]. Like it has been pointed out by other study groups, there is a significant correlation between the time needed to complete the report and the complexity of the pathology described [26]. While unremarkable or common pathological findings are quickly assessed using SRs, complex pathologies tend to be time consuming. This is mostly caused by the high number of elements needed within the template and the need to use free text elements which have been proven to be the most time-consuming [10, 38]. However, rapidity in generating FTRs might be due to the fact that these reports are significantly inferior in overall report quality, completeness and readability.

When comparing the time required to generate FTRs and SRs, several other effects have to be taken into account: Every change in the workflow will result in an initial loss of time due to the introduction of a new method, since most physicians are currently trained for FTRs. Therefore, studies are likely to assess this initial loss of time and not the resulting speed-up in the long term. One further aspect is the effect of writing more comprehensive reports. Radiologists as well as pathologists struggle with large numbers of follow-up queries due to ambiguous or incomplete reports. A recent survey about the introduction of synoptic reporting in cancer pathology in different countries evaluated this question [39]. The authors concluded that the additional time spent on SRs is exclusively seen in the beginning and that implementation actually resulted in a significant reduction of time needed to complete reports. Therefore, it is also likely for other disciplines that introducing synoptic reporting will also be time-efficient in the long run. The integration of structured reporting into pre-existing clinical information systems will be the next milestone [40]. Furthermore, interviewed examining physicians stated unanimously that even though SRs tend to be more time-consuming, the additional time needed (+ 69.2 s, *p* < 0.001) is well spent due to the significantly increased report quality (+ 49.3%, *p* < 0.001), level of detail of pathologies (+ 36.6%, *p* < 0.001) and readability (+ 52.9%, *p* < 0.001). This may be emphasized by taking into account that report content is the base for clinical decisions [9]. Whether the increased report quality of SRs is associated with a more sophisticated therapy or even with a better outcome has to be answered by future studies.

## Conclusions

In conclusion, structured reporting is a solid approach to generate high quality, detailed and comparable reports. The additional time needed to complete the report is acceptable with regard to the superior clarity of the report and does not impair clinical workflow efficiency. Examiners and the referring physicians have a significant preference for SRs of head and neck ultrasound examinations. Our data suggest that SRs of head and neck ultrasound examinations should be the standard report in clinical practice and scientific work.

## Abbreviations

FTR: Free text report
SR: Structured report
VAS: Visual analog scale
